# Perineural Capsaicin Treatment Inhibits Collateral Sprouting of Intact Cutaneous Nociceptive Afferents

**DOI:** 10.3390/biomedicines10061326

**Published:** 2022-06-04

**Authors:** Péter Sántha, Szandra Lakatos, Ágnes Horváth, Mária Dux, Gábor Jancsó

**Affiliations:** 1 Department of Physiology, University of Szeged, Dóm tér 10, 6720 Szeged, Hungary; lakatos.szandra@med.u-szeged.hu (S.L.); dux.maria@med.u-szeged.hu (M.D.); 2 1st Department of Internal Medicine, University of Szeged, Korányi fasor 8-10, 6720 Szeged, Hungary; horvath.agnes.judit@gmail.com

**Keywords:** collateral sprouting, neurogenic inflammation, capsaicin TRPV1, TRPA1, scanning laser Doppler perfusion imaging

## Abstract

Perineural treatment of peripheral nerves with capsaicin produces a long-lasting selective regional thermo- and chemo-analgesia and elimination of the neurogenic inflammatory response involving degeneration of nociceptive afferent fibers. In this study, we examined longitudinal changes in mustard oil–induced sensory neurogenic vasodilatation and plasma extravasation following perineural capsaicin treatment of the rat saphenous nerve utilizing scanning laser Doppler imaging and vascular labeling with colloidal silver. Capsaicin treatment resulted in a marked decrease in mustard oil–induced vasodilatation in the skin area served by the saphenous nerve. Repeated imaging of the vasodilatatory response showed no recovery for at least 7 weeks. However, following transection and ligation of the capsaicin-treated saphenous nerve, a substantial recovery of the vasodilatatory response was observed, suggesting a reinnervation of the chemodenervated skin area by collateral sprouting of neighboring intact sciatic nerve afferents. Elimination of the recovered vascular reaction by capsaicin treatment of the sciatic nerve supported this conclusion. Similar results have been obtained by using the vascular labeling technique. These findings indicate an inhibitory effect of persisting cutaneous nerve fibers on the collateral sprouting of intact nerve fibers into the chemodenervated skin area. These observations may bear implications for the development of sensory disturbances following peripheral nerve injuries.

## 1. Introduction

Injuries inflicted upon peripheral nerves result in the immediate functional deterioration of sensory functions often associated with short-term degenerative and delayed regenerative processes. Damage to peripheral nerves may cause either an indiscriminate lesion in all types of nerve fibers or may be directed towards a specific class or classes of axons. Nerve transection and crush produce loss of function in all nerve fibers running in the affected nerve, whereas in metabolic (diabetes) or toxic (alcohol, antitumor drugs, antibiotics, toxic metals) neuropathies specific classes of axons may be affected [1,2,3]. Capsaicin, the pungent agent in red peppers, is a selective neurotoxin acting through the transient receptor potential vanilloid type 1 receptor (TRPV1), selectively targeting C-fiber nociceptive afferent axons [4]. Local application of capsaicin and related vanilloids onto peripheral nerve trunks produces a selective defunctionalization and/or chemodenervation of nociceptive afferent axons, resulting in an apparently permanent regional nociceptor analgesia characterized by a loss of sensitivity to pain-producing chemical irritants, profound decrease in heat-pain sensitivity and the abolition of the neurogenic inflammatory response strictly confined to the innervation territory of the affected nerve [5,6,7,8,9,10]. Immunohistochemical and quantitative electron microscopic findings revealed that this may be, at least in part, accounted for by a loss of unmyelinated, C-fiber afferent axons in the capsaicin-treated nerve and the skin area innervated by that nerve [7,11,12]. It was also shown that, unlike after nerve transection or crush lesions [13,14], the denervated skin area showed no signs of functional reinnervation by regenerative or collateral sprouting of the affected or the adjacent intact nerves, respectively [15]. However, due to the lack of techniques capable of a quantitative, longitudinal evaluation of the process of functional reinnervation of the denervated skin by peptidergic nociceptive C-fibers, the temporal and spatial patterns of degenerative and regenerative processes could not be investigated. Recently, we have developed a novel experimental approach which permits the longitudinal assessment of the functional regeneration of peptidergic nociceptive afferents following peripheral nerve transection. The technique is based on the assessment of the intensity and spatial distribution of mustard oil–induced changes in skin blood flow by utilizing scanning laser Doppler imaging [16]. Mustard oil is an agonist of transient receptor potential ankyrin type 1 (TRPA1) receptors [17], which are expressed in all TRPV1 receptor–expressing nociceptive primary sensory neurons [18,19]. Importantly, mustard oil has also been shown to be a potent activator of the TRPV1 receptor [20]. Activation of these receptors by mustard oil results in the release of the neuropeptides calcitonin gene–related peptide (CGRP) and substance P from sensory nerve endings, which in turn elicit neurogenic sensory vasodilatation and plasma extravasation, collectively termed the neurogenic inflammatory response, a salient feature of peptidergic nociceptive afferent function [21,22,23,24]. Previous studies have shown that skin areas delineated by mustard oil–induced increased vascular permeability as assessed with the Evans blue method [14,15,25,26,27] or the vascular labeling technique [7] correspond to the innervation territories of nerve endings stimulated by antidromic electrical nerve stimulation or the direct application of chemical irritants onto the skin. Importantly, cutaneous areas displaying sensory nerve stimulation–induced vasodilatation and plasma extravasation are strictly coincident with the innervation territory of the stimulated nerve [7,16,28]. Therefore, topographical localization of mustard oil–induced vasodilatation and plasma extravasation can be used to assess the pattern of cutaneous innervation in naïve and nerve-injured animals [14,15,16,26].

The present study was initiated to elucidate the regenerative propensity of intact and capsaicin-treated nerves serving the dorsum of the rat hind paw through longitudinal examination of changes in the topography and intensity of mustard oil–induced sensory neurogenic vasodilatation and plasma extravasation in the rat. Application of the novel experimental paradigm utilizing scanning laser Doppler imaging was expected to reveal the time course and mechanisms of collateral sprouting of cutaneous afferents following a specific chemical lesion of nociceptive sensory neurons. The findings may also be of interest with respect to the possible therapeutic application of TRPV1 agonists for pain relief.

## 2. Materials and Methods

Experiments were performed on adult male Wistar rats (*n* = 18) weighing 250–280 g at the beginning of the experiments. The animals were kept in a 12 h light/dark cycle in standard cages with free access to food and water. All experiments were carried out according to the guidelines of the European Communities Council Directive of 24 November 1986 (86/609/EEC) and the Council Regulation 40/2013 (II. 14.). The experimental protocol was reviewed by the Ethics Committee for Animal Care at the University of Szeged. The number of experimental animals was kept as low as possible.

### 2.1. Surgery

Rats were anaesthetized with an intraperitoneal injection of a combination of ketamine (Calypsol, 70 mg/kg, Gedeon Richter, Budapest, Hungary) and xylazine (CP-Xylazin 2%, 10 mg/kg, Produlab Pharma, Raamsdonksveer, The Netherlands). Animals were placed on a heating pad to keep their body temperature at a constant level of 37 ± 0.5 °C. Room temperature was kept at 22–23 °C.

### 2.2. Perineural Capsaicin Treatment

**Figure 1:**
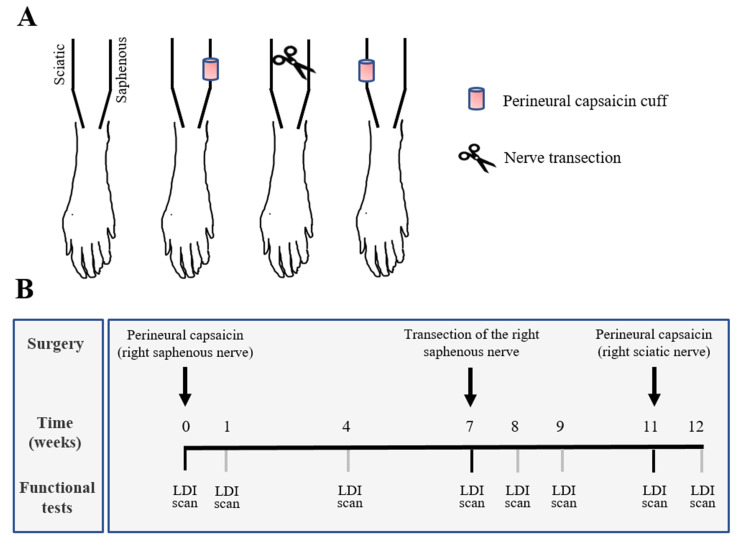
(**A**) Schematic illustration of the types and sequence of surgical interventions applied in this study. (**B**) Timeline of the experiments illustrating the sequence of surgical interventions and functional testing with laser Doppler imaging (LDI) following perineural capsaicin treatment of the saphenous nerve.

The right saphenous nerve was exposed in the thigh, isolated from the surrounding tissues with Parafilm^®^ (Sigma-Aldrich, St. Louis, MO, USA) and a small piece of gel foam soaked with 0.1 mL of a 1% solution of capsaicin (Sigma-Aldrich, St. Louis, MO, USA; dissolved in saline containing 6% ethanol and 8% Tween 80) was applied around the nerve. After 20 min, the gel foam was removed, the wound was closed, and the rat was returned to the animal cage (Figure 1A).

### 2.3. Peripheral Nerve Transection

After a follow-up period of 7 weeks, the previously capsaicin-treated right saphenous nerve was exposed high in the thigh and transected distal to a ligature (Figure 1A). To prevent regeneration of the nerve, a 0.5 cm long segment of the distal stump was removed. The wound was then closed and the rat was returned to the animal cage.

### 2.4. Measurement of Cutaneous Blood Flow with Scanning Laser Doppler Flowmetry

Rats were anaesthetized with a combination of ketamine (Calypsol, 70 mg/kg) and xylazine (CP-Xylazin 2%, 10 mg/kg). Scanning laser Doppler flowmetry using a PeriScan PIM3 scanning laser Doppler imager (Perimed, Järfälla, Sweden) was utilized to determine changes in the intensity and topographical distribution of chemically-induced increases in cutaneous blood flow following peripheral nerve lesions. The dorsal surface of both hind paws was scanned by using the repeated scan mode with 52 × 42 pixel frame size. Distance of the scanner aperture from the skin surface was set to 19 cm and the laser beam was perpendicular to the skin surface. Perfusion images were captured every 2 min and measurements took 15–20 min for each animal. All flow values were expressed as means ± S.D. Basal tissue perfusion and changes in blood flow induced by painting the skin of the dorsum of the hind paw with mustard oil (5% in liquid paraffin) were recorded in arbitrary perfusion units (PU) and expressed as per cent change relative to baseline. The linear velocity values and the concentration of moving erythrocytes in the skin volume fraction detected by the scanner at any instances are combined in the PU value [29]. The average of three subsequent measurements before the application of mustard oil was calculated to determine baseline value. Images displaying the maximum vasodilatatory responses were used in each experiment to quantitatively evaluate the reactions. Scanning laser Doppler images were taken before surgery and 1–7 weeks after perineural capsaicin treatment of the saphenous nerve. Additional measurements were made 1–4 weeks after transection of the saphenous nerve previously treated with capsaicin (Figure 1B).

### 2.5. Evaluation of Perfusion Data

The innervation territory of the saphenous nerve was defined on the basis of perfusion images taken 1 week after perineural capsaicin treatment of the saphenous nerve. Color-coded perfusion images representing the maximal vasodilatation were further processed with the ImagePro 6.2 software (MediaCybernetics, Rockville, MD, USA). Changes in the intensity of the vasodilatatory responses measured in the saphenous and sciatic skin areas were considered as evidence of functional reinnervation.

### 2.6. Separation of Innervated and Denervated Skin Areas

The color-coded perfusion images showing the maximal vasodilatatory response were used to determine the proportion of innervated and denervated skin areas, respectively. Data of all pixels were normalized to the 90th percentiles of the maximal response, then the 20th percentile of the lateral (intact) side was calculated to define a threshold value for each individual image. Pixels with data lower than the 20th percentiles of the intact side were considered as denervated to separate areas showing no or minimal increase in vasodilation from those exhibiting large (or maximal) perfusion increases. This step was followed by the generation of a binary mask representing the size and topography of denervated cutaneous areas corresponding to the innervation area of the saphenous nerve.

### 2.7. Vascular Labeling Technique

To study the size and topographical distribution of the innervated and denervated skin areas, another functional-morphological method, the vascular labeling technique was also used. This technique enables the visualization of small blood vessels of increased permeability to colloid particles. Hence, vascular labeling is a salient feature of the (neurogenic) inflammatory response [25,30]. Vascular labeling experiments were performed 4 weeks after perineural capsaicin treatment of the saphenous nerve, and 2, 3 and 4 weeks after transection of that same nerve. Briefly, the anesthetized animals were injected intravenously with 1% solution of colloidal silver (Sigma-Aldrich, St. Louis, MO, USA; 50 mg/kg b.w.) and the right hind paw skin was treated with 5% mustard oil solution immediately after completion of the injection. The vascular labeling of small blood vessels (venules) exhibiting increased permeability was observed as intense brownish color of the innervated skin in sharp contrast with the denervated skin regions, where the reaction is absent. Close-up photographs were made to document the extension of cutaneous vascular labeling. The size of the total skin area of the hind paw and the proportions of the innervated and denervated skin areas were determined with planimetry using ImagePro software. To visualize the topographical distribution of vascular labeling under the light microscope, the dorsal skin of the hind paw was removed, flattened, dehydrated in graded alcohols and made transparent with xylene. The specimens were mounted on glass slides and cover-slipped with Canada balsam.

### 2.8. Statistics

Data represent means ± S.D. of 4–9 independent measurements. For statistical comparisons of the mustard oil–induced vasodilatatory responses and the change in the proportions of innervated and denervated skin areas, respectively, the one-way ANOVA test was performed followed by multiple comparisons using Fisher’s least significant difference test (time course of mustard oil–induced vasodilation) or Dunnett’s post hoc analysis. In all groups, normality was proved by the Shapiro–Wilk test and homogeneity of variances was confirmed by Levene’s test in advance of performing ANOVA. Statistical analysis was performed by using Statistica 13.0 software (Dell Inc., Tulsa, OK, USA).

## 3. Results

### 3.1. Effect of Perineural Capsaicin Treatment of the Saphenous Nerve on Mustard Oil–Induced Neurogenic Vasodilatation in the Rat Hindpaw

**Figure 2:**
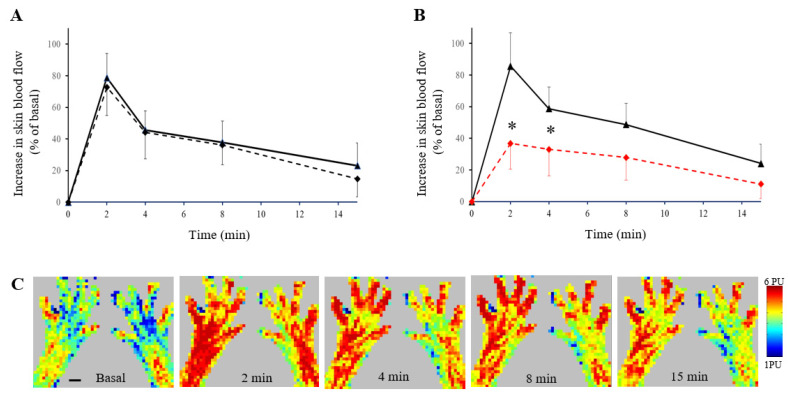
(**A**) Time course of mustard oil–induced increases in perfusion of the medial (dashed line) and lateral (solid line) parts of the dorsal skin of the right hind paws of rats (*n* = 5). (**B**) Time course of mustard oil–induced increases in perfusion of the medial (dashed red line) and lateral (solid line) parts of the dorsal skin of the right hind paws of rats 1 week after perineural treatment of the right saphenous nerve (*n* = 5). *: significantly different from the perfusion of the lateral part of the dorsum of the hind paw skin. (**C**) A series of perfusion images illustrating the time course of mustard oil–induced increases in blood flow in the dorsum of a rat hind paw. The right saphenous nerve was treated with capsaicin 5 days before the experiment. Scale bar represents 5 mm.

In accord with previous findings, changes in cutaneous blood flow could be reliably demonstrated with scanning laser Doppler imaging. Application of mustard oil onto the dorsum of the intact rat hind paw resulted in an increase in skin perfusion which reached its maximum after 2 min and amounted to 78.76 ± 15.42 and 72.64 ± 17.85 percent of the basal value in the lateral and medial parts, respectively, of the dorsal skin of the right hind paw. The vasodilatatory response gradually subsided and returned to the initial basal value after about 15 min (Figure 2A). One week after perineural capsaicin treatment of the right saphenous nerve, the dorsal skin of the intact (left) hind paw and the lateral part of the dorsal skin of the right hind paw displayed marked increases in blood flow. In contrast, in the medial part of the dorsal skin of the righthind paw, mustard oil–induced increase in perfusion was markedly and significantly reduced amounting only to 39.03 ± 16.38 percent of the basal value (Figure 2B). A series of perfusion images taken from the dorsal skin of the hind paws 1 week after perineural capsaicin treatment of the right saphenous nerve illustrates these changes (Figure 2C). These findings demonstrate that perineural capsaicin treatment of the saphenous nerve resulted in an impairment of the “efferent” vasodilatatory function of peptidergic afferent nerves which innervate the medial aspect of the dorsum of the rat hind paw.

### 3.2. Effect of Transection of the Capsaicin-Treated Saphenous Nerve on Mustard Oil–Induced Neurogenic Vasodilatation in the Rat Hindpaw

**Figure 3:**
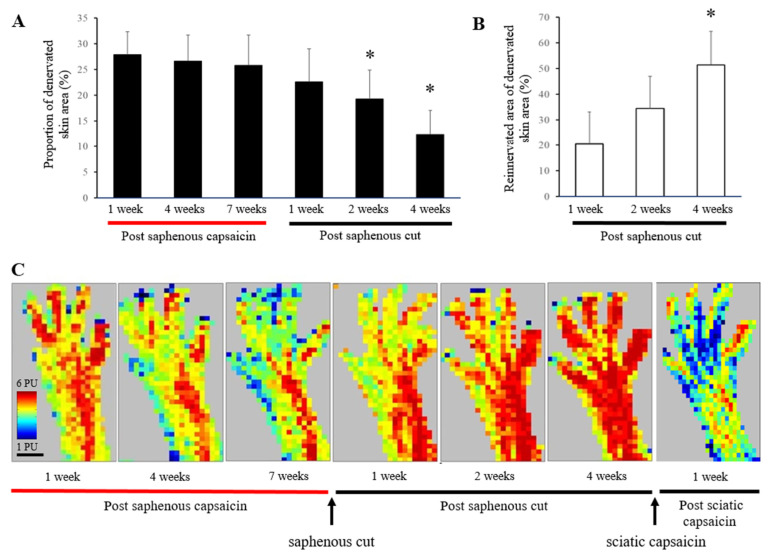
(**A**) Calculation of denervated skin areas following perineural capsaicin treatment of the saphenous nerve and subsequent transection of that nerve 7 weeks later (*n* = 9). *: significantly different from the skin area determined 1 week after perineural capsaicin treatment. (**B**) Calculation of reinnervated skin areas following transection of the saphenous nerve treated perineurally with capsaicin 7 weeks previously (*n* = 7). *: significantly different from the denervated area determined 1 week after perineural capsaicin treatment. (**C**) A series of laser Doppler scanning perfusion images of the rat hind paw following perineural capsaicin treatment of the saphenous nerve and subsequent transection of the saphenous nerve and perineural capsaicin treatment of the sciatic nerve 7 and 11 weeks later, respectively. Scale bar represents 5 mm.

Figure 3A shows that there is no significant change in the proportion of denervated skin areas up to 7 weeks after perineural capsaicin treatment. Since no signs of functional restitution was observed even 7 weeks after perineural capsaicin, it can be assumed that the marked and permanent reduction of the vasodilatatory response may result from degenerative changes in the affected saphenous nerve afferents serving the medial part of the dorsum of the hind paw. Hence, innervated and denervated skin areas of the dorsal hind paw skin were delineated as described in the Methods section. Further, we speculated that nerve fibers other than peptidergic capsaicin-sensitive afferents, which persist in capsaicin-treated nerve and skin, may impede collateral invasion of intact afferent nerve fibers from the neighboring sciatic innervation territory of the skin. Therefore, we transected the (right) saphenous nerve 7 weeks after capsaicin treatment and measured the vasodilatatory responses thereafter. Evaluation of perfusion images disclosed a gradual decrease in the proportion of denervated skin areas 2–4 weeks after transection of the saphenous nerve (Figure 3A). Accordingly, in the chemodenervated skin area, the proportion of reinnervated areas gradually increased and after 4 weeks peaked at around 40 percent (Figure 3B). The temporal and spatial characteristics of the effect of perineural capsaicin treatment of the saphenous nerve and the reinnervation process of the chemodenervated area are illustrated in the series of perfusion images in Figure 3C.

These findings suggest a reinnervation of the chemodenervated skin by collateral sprouting of intact sciatic afferents, provided that the capsaicin-treated saphenous nerve is transected. This is supported by the finding that perineural capsaicin treatment of the sciatic nerve abolished the recovered vasodilatatory response within the confines of the capsaicin-treated saphenous nerve (Figure 3C).

### 3.3. Effect of Perineural Capsaicin Treatment of the Saphenous Nerve on Mustard Oil–Induced Vascular Labeling in the Rat Hindpaw

**Figure 4:**
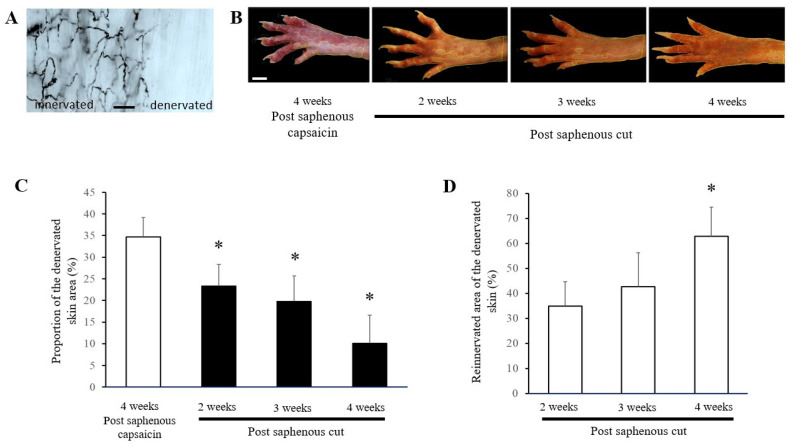
(**A**) A microphotograph illustrating vascular labeling of the hind paw skin of a rat following an intravenous injection of a colloidal silver solution. Colloidal silver–labeled small blood vessels (venules) are present in the innervated but not the denervated skin. (**B**) A series of photographs taken from the right hind paw of a rat to illustrate the appearance of mustard oil–induced vascular labeling in the innervated skin areas 4 weeks after perineural capsaicin treatment of the saphenous nerve and after a subsequent transection of that nerve. Scale bar represents 5 mm. The quantitative data showing the proportions of the denervated and reinnervated skin areas are shown in (**C**) (*n* = 4, *: significantly different from the skin area determined 4 weeks after perineural capsaicin treatment) and (**D**) (*n* = 4, *: significantly different from the skin area determined 4 weeks after perineural capsaicin treatment), respectively.

In accord with earlier reports, cutaneous application of mustard oil produces a clear-cut vascular labeling of small blood vessels (venules) in innervated but not chemodenervated skin areas following an intravenous injection of colloidal silver [7,16]. This is illustrated in Figure 3A. Importantly, labeling by colloidal silver can also be observed macroscopically, photographed and the innervated and denervated areas measured with a computer program. The results obtained with the vascular labeling technique and scanning laser Doppler imaging were essentially similar. The size and topography of the denervated skin area, as well as the gradual recovery of the vascular labeling, is illustrated in a series of photographs taken at different time intervals after perineural capsaicin treatment and subsequent transection of the saphenous nerve (Figure 4B). The quantitative data show a substantial reinnervation of the chemodenervated skin following transection of the capsaicin-treated (saphenous) nerve. Hence, the proportion of the chemodenervated skin area gradually decreased, whereas in the chemodenervated skin area the proportion of the reinnervated territory gradually increased after transection of the capsaicin-treated saphenous nerve (Figure 4C,D).

## 4. Discussion

Perineural treatment with capsaicin or other vanilloids has been shown to produce marked nociceptor analgesia, i.e., chemo- and thermo-analgesia and inhibition of neurogenic plasma extravasation confined to the innervation territory of the treated nerve [5,6,7,8,9,10,31]. The effect of capsaicin on peripheral nociceptive C-fibers is mediated by TRPV1 [32] and is associated with a decrease in afferent nerve fibers. Quantitative electron microscopic studies on rat saphenous nerves treated with capsaicin revealed a 32–36 percent loss of unmyelinated axons [11,12]. This indicates that not all capsaicin-sensitive afferent axons are lost after perineural treatment with capsaicin, since the proportion of capsaicin-sensitive afferents amount to 64–70 percent in the saphenous nerve [33,34]. Collectively, these observations suggested that, following perineural treatment with capsaicin, the loss of function of capsaicin-sensitive afferents may be accounted for by both a loss of unmyelinated axons [7,11,12] and depletion of vasoactive neuropeptides substance P and CGRP from capsaicin-sensitive cutaneous afferents [7,31].

The possible restitution of the function of peptidergic afferents in a skin area chemodenervated by perineural application of capsaicin has been examined in previous studies by making use of neurogenic plasma extravasation to study cutaneous nerve regeneration. These experiments demonstrated a complete disappearance of neurogenic plasma extravasation in the cutaneous innervation territories served by the affected nerve. In contrast to peripheral nerve transection or crush, no tendency for recovery of the vascular response was observed [15]. A significant drawback to the application of the Evans blue–based technique for the demonstration of cutaneous plasma extravasation is that it is unsuitable for longitudinal studies in the same animal.

In the present study, the experimental paradigm based on scanning laser Doppler imaging of mustard oil–induced neurogenic sensory vasodilatation in the rat hind paw permitted the longitudinal examination of changes in the vasodilatatory response, i.e., the putative functional regeneration of peptidergic, in particular the CGRP-containing sensory nerves which mediate this vascular reaction [22,23,24]. This approach has recently been successfully used to reveal the time course and mechanisms of the functional regeneration of peptidergic cutaneous nerves following neurotmesis of the saphenous nerve in the rat. It was demonstrated that transection and ligation of the saphenous nerve resulted in a substantial reduction of the vasodilatatory response in the innervation territory of the nerve, which, however, displayed a gradual, albeit not complete, recovery towards control after 4–6 weeks. Evidence was presented that this recovery resulted from collateral sprouting of intact sciatic afferents into the denervated saphenous skin area [16].

Perineural treatment of the saphenous nerve with capsaicin resulted in a complete and permanent loss of mustard oil–induced neurogenic sensory vasodilatation in the skin area served by that nerve. Further, no tendency for recovery of the vasodilatatory response was observed up to a survival period of 7 weeks, suggesting that neither the afferents running in the treated saphenous nerve nor the intact sciatic afferents innervating the adjacent skin area invaded the denervated skin via regenerative or collateral sprouting, respectively.

The most intriguing finding of the present study is, however, the restoration of the regenerative propensity of intact nociceptive axons of the sciatic nerve following the transection of the capsaicin-treated saphenous nerve. Indeed, in the chemodenervated skin area, a gradual and significant recovery in both intensity and the spatial extent of the mustard oil–evoked vasodilatatory response was observed following transection (and ligation) of the capsaicin-treated saphenous nerve. Since the saphenous nerve was prevented from regeneration, this finding indicates an invasion of adjacent intact sciatic nerve afferents into the denervated saphenous skin territory, reinnervating the denervated area by way of collateral sprouting, similarly to that observed after transection and ligation of the saphenous nerve [16]. This assumption was strongly supported by the elimination of the vasodilatatory response in skin areas served by the sciatic and by the saphenous nerve after perineural treatment of the sciatic nerve with capsaicin. This indicates that the vasodilatatory response observed in the saphenous skin area may be indeed attributed to activation of sciatic afferents which sprouted into the saphenous skin area.

The results obtained by making use of the phenomenon of mustard oil–induced vascular labeling to delineate innervation territories of peripheral nerves yielded similar results. Previous light microscopic studies demonstrated that skin areas exhibiting vascular labeling strictly coincide with skin areas of intact peptidergic sensory innervation [7]. Therefore, measurement of silver-stained skin regions furnishes reliable information on the functional integrity of cutaneous peptidergic nerves. In naïve rats, application of mustard oil onto the dorsal skin of the hind paw produced a brownish coloration of the skin, indicative of increased vascular permeability as a result of neurogenic inflammation. Perineural capsaicin treatment of the saphenous nerve resulted in the complete abolition of the mustard oil–induced brownish coloration of the skin area served by that nerve. Repeated measurements failed to indicate significant changes in silver-stained skin areas for at least 4 weeks. However, transection and ligation of the saphenous nerve resulted in gradual increase in the silver-stained skin area, indicating the recovery of function of the sensory nerves mediating the neurogenic inflammatory response. Since the saphenous nerve was transected and ligated, this may result from collateral sprouting of intact sciatic afferents serving adjacent skin areas. This conclusion is supported by the observation showing a complete abolition of silver-containing skin areas following perineural capsaicin treatment of the sciatic nerve.

Collectively, the present findings confirmed previous observations which demonstrated a lack of regeneration of afferent nerve fibers following perineural treatment with capsaicin and, importantly, the failure of the collateral sprouting of intact sensory fibers into the chemodenervated skin area [15]. Importantly, however, the present experiments demonstrated that transection and ligation of the nerve treated previously with capsaicin restores the propensity of intact nerve fibers for collateral sprouting.

These observations suggest a novel mechanism promoting peripheral nerve regeneration, in particular collateral sprouting of nociceptive afferents. However, the present experiments provide no clues as to the mechanism(s) of this permissive conditioning lesion effect promoting collateral sprouting. Mechanistically, non-degenerating capsaicin-sensitive and intact unmyelinated and myelinated nerve fibers which persist in the chemodenervated skin may inhibit the invasion of adjacent intact sciatic nerve fibers into a denervated skin area. Collateral sprouting of intact sensory axons is critically dependent on the production of trophic factors and/or cytokines in degenerating nerves [35] or denervated target tissue [36] triggered by degenerating axons. Moreover, discordant changes in the expression of cytokines and chemokines were demonstrated following peripheral nerve transection and perineural treatment with capsaicin [37]. The lack of Wallerian degeneration of myelinated and capsaicin-insensitive unmyelinated afferent and sympathetic axons following the vanilloid treatment of peripheral nerves [9] and consequent failure of the production of specific signaling molecules may be a likely explanation for the failure of the collateral sprouting of intact sciatic afferents. The induction, by transection of the nerve previously treated with capsaicin, of Wallerian degeneration of cutaneous nerve fibers spared by capsaicin-induced chemodenervation may lift this inhibitory influence by creating favorable tissue microenvironment for promoting invasion of afferent axons from neighboring skin of intact innervation. Further studies on the molecular mechanisms of this permissive conditioning lesion effect may promote our understanding of the regulation of axonal regenerative processes under neuropathic conditions characterized by the partial sparing of nerve fibers and target innervation, such as diabetic or toxic neuropathies.

## Data Availability

Not applicable.
